# Repurposing dipeptidyl peptidase-4 inhibitor for Parkinson's disease prevention: A drug-target Mendelian randomization study

**DOI:** 10.1016/j.neurot.2025.e00815

**Published:** 2025-12-29

**Authors:** Joo-Yeon Lee, Don Gueu Park, Joohon Sung

**Affiliations:** a Institute of Health and Environments, Seoul National University, Seoul, 08826, South Korea; b Ajou University School of Medicine, Suwon, Gyeonggi-do, 16499, South Korea; c Department of Epidemiology, Graduate School of Public Health, Seoul National University, Seoul, 08826, South Korea; d Genome Medicine Institute, Seoul National University Medical Research Center, 03080 Seoul, South Korea

**Keywords:** Mendelian randomization, Parkinson's disease, Drug repurposing, Dipeptidyl peptidase-4 inhibitor

## Abstract

Parkinson's disease (PD) remains without effective disease-modifying therapies, primarily due to limited understanding of its underlying mechanisms. Emerging evidence suggests that dipeptidyl peptidase-4 inhibitors (DPP-4Is), commonly used as anti-diabetics, may offer neuroprotective effects. We aimed to evaluate the potential of repurposing DPP-4Is for reducing the PD risk, using a drug-target Mendelian randomization (MR) approach, with specific attention to sex. We performed two-sample MR analyses using instrumental variables (IVs) for DPP-4Is derived from gene expression quantitative trait loci (IV_eQTL_) and protein QTL (IV_pQTL_) data sourced from the eQTLGen consortium and UK Biobank, respectively. Associations were tested using the largest PD genome-wide association studies (GWAS) dataset from the International Parkinson's Disease Genomics Consortium, including sex-stratified analyses. Primary analyses used inverse variance weighted MR, with additional sensitivity analyses (alternative MR methods, varying IV selection thresholds, and tissue-specific eQTL), mediation analysis through diabetes and colocalization analysis. Genetically proxied DPP-4 inhibition was associated with reduced PD risk: OR (95% CI) ​= ​1.78 (1.21–2.61) for IV_eQTL_ and 1.21 (1.02–1.45) for IV_pQTL_. This beneficial effect was more pronounced in men—IV_eQTL_: 2.25 (1.55–3.28); IV_pQTL_ 1.35 (1.15–1.57)—but not significant in women, suggesting sex-specific effects. Findings were robust across sensitivity analyses and replicated in an independent PD GWAS. Diabetes did not mediate this relationship, and colocalization provided partial evidence for a shared causal variant only in men. This multi-omics drug-target MR study suggests that DPP-4Is may reduce PD risk, supporting their potential for repurposing, particularly in male patients.

## Graphical abstract

**Figure:**
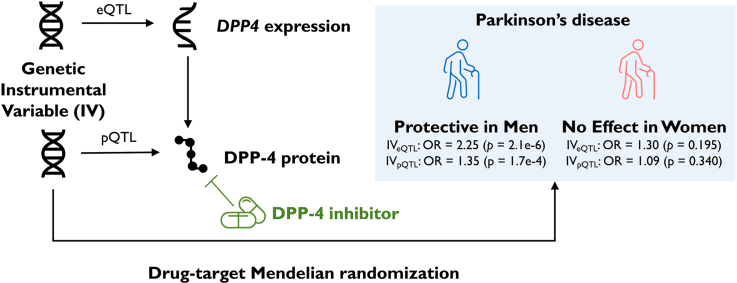

## Introduction

Parkinson's disease (PD) is a progressively debilitating neurodegenerative disorder with its pathogenesis still not fully understood, impeding the discovery of disease-modifying treatments. Recent studies have identified promising therapeutic targets, with epidemiological evidence suggesting potential protective effects of anti-diabetic drugs, specifically glucagon-like peptide-1 receptor agonists (GLP-1RA) and dipeptidyl peptidase-4 inhibitors (DPP-4Is), on PD risk [[1], [2], [3]]. Notably, GLP-1RAs demonstrated beneficial effects in phase 2 clinical trials for PD [4,5]. Although a recent phase 3 trial of GLP-1RA on PD failed to meet its primary endpoint, it demonstrated favorable trends across secondary outcomes [6]. Moreover, both GLP-1RAs and DPP-4Is have been associated with neuroprotective effects in other neurodegenerative diseases, including Alzheimer's disease [[7], [8], [9]]. Although the mechanism of DPP-4Is partly overlap with GLP-1RA, such as increasing incretin action [10], but its potential for PD management remains unconfirmed, as no clinical trials have been completed to date. Unlike GLP-1RA, predominantly administered parenterally, DPP-4Is are available as oral agents.

Mendelian randomization (MR) analyses have emerged as a powerful tool for inferring causal relationships between risk factors and health outcomes using genetic instrumental variables (IVs). Drug-target MR, which constructs genetic proxies for drug targets, offers promising approach for identifying novel molecular targets and repurposing existing drugs. The advancement of multi-omics data has further enhanced the accuracy of these genetic proxies, leveraging the gene-expression data. Genetic variants for IVs are further enriched by including expression quantitative trait loci (eQTL) or protein QTL (pQTL) data, to represent drug impacts. Results of MR studies have aligned with clinical trial results, both showing, for example, that increasing HDL levels has a null effect on coronary artery disease risk, contrary to observational studies [11,12]. Drug-target MR for DPP-4I would provide evidence that may predict the results in clinical trial.

PD exhibits a sex-related disparity, with males showing approximately 1.5 times higher prevalence than females [13], and divergent disease progression and complications between sexes [14,15]. These sex-specific characteristics underscore the complexity of PD's underlying mechanisms, highlighting the importance of sex-stratified analyses in PD.

This study aims to evaluate the potential for repurposing DPP-4Is for preventing PD using a drug-target MR approach. We employ IVs derived from eQTL and pQTL data to investigate the causal relationship between DPP-4 inhibition and PD risk, overall and by sex. Furthermore, we examined our results using eQTL data from various tissues and in relation to prodromal stage of PD. By leveraging these diverse methodologies, we seek to provide novel insights into the sex-specific therapeutic potential of DPP-4Is in PD prevention.

## Materials and Methods

### IV Development

In constructing the genetic IV for DPP-4, we employed the eQTL results from the eQTLGen consortium (phase 1), which incorporated 37 eQTL data from 31,684 samples of predominantly European ancestry [16]. We only extracted significant *cis*-eQTL loci located within ±200 ​kb of the gene boundary, applying Bonferroni correction (*p*-value <0.05/number of markers). From these data, we included SNPs with no linkage disequilibrium (r^2^ ​< ​0.1) to generate the IVs.

To capture the influence of drug targets on downstream biological components, we also constructed genetic instruments related to plasma protein levels using data from a recent genome-wide association study (GWAS) of proteome in the UK Biobank [17]. We identified significant *cis*-pQTLs (within ±200 ​kb) with *p*-values <0.05/number of markers. Both eQTL and pQTL results were derived using normalized data and beta estimates of each SNP on this normalized value were taken in our MR analysis.

### Association with the PD risk (Primary MR analysis)

With the diverse IVs generated using gene expression (IV_eQTL_) or protein (IV_pQTL_) data, we assessed their impact on PD risk. We utilized data from the International Parkinson's Disease Genomics Consortium (IPDGC) which conducted GWAS on 37,688 PD patients and 981,372 controls, predominantly of European ancestry [18]. When we estimated the mean age weighted by the standard error within each study, including only studies where age data were available, the mean age was 61.7 years in cases and 64.5 years in controls, respectively [18]. For sex-stratified analysis, we used the data from PD GWAS conducted by sex within the same consortium, including 12,054 PD cases and 11,999 controls in men, and 7,384 PD cases and 12,389 controls in women [19]. For the replication phase, meta-analysis of PD in UK Biobank [20] and FinnGen [21] was used, which included 7,521 cases and 897,736 controls. Results of both sexes combined were used since sex-stratified results were not available.

MR analysis was conducted using the inverse variance weighted (IVW) method or a Wald ratio if there was only one SNP in the IV, utilizing the ‘TwoSampleMR’ package (v0.5.8) in R [22]. This research followed the STROBE-MR statement as detailed in the Supplement [23].

### Assessment of IV assumptions

We evaluated three core IV assumptions (relevance, independence, and exclusion restriction assumptions) as follows. To ascertain the robustness of each IV (relevance assumption), we calculated their *F* statistics using the following formula:

(1) $$\left[\left(N-K-1\right)/K\right]\times \left[R^{2}/\left(1-R^{2}\right)\right]$$

where *N* and *K* denote the sample size and number of SNPs comprising the IV, respectively. The variance explained by each SNP (*R*^*2*^) was computed using the effect estimate (*β*) and the effect allele frequency (*EAF*), based on the equation [2].

(2) $$2\times \beta^{2}\times \text{EAF}\times \left(1-\text{EAF}\right)$$

The independence assumption that the SNPs used as instruments for the exposure (DPP-4 inhibition) are not associated with confounders was not evaluated, since we used the summary-level data. However, IVs specifically mimicking a drug target gene usually consist of very small number of SNPs (within ±200 ​kb of *DPP4* in this study), thereby violation of second assumption is unlikely. The exclusion restriction assumption (no pleiotropy) was evaluated with the MR-Egger analysis.

### Sensitivity analyses

We assessed for heterogeneity and pleiotropy in all MR analyses. Upon detecting heterogeneity using Cochran's Q statistic (*p* ​< ​0.05), outliers were removed for re-analysis utilizing the ‘RadialMR’ software. We employed the MR-Egger estimates when horizontal pleiotropy was detected (*p* ​< ​0.05). We also performed the MR-Steiger directionality test [22] to confirm the direction of causality between *DPP4* expression or protein levels and PD. Statistical power was calculated with mRnd (https://shiny.cnsgenomics.com/mRnd/) for each MR analysis [24]. Analysis of IV robustness was further tested by employing varied LD clumping thresholds (*r*^*2*^ ​< ​0.3, 0.2, 0.05, and 0.01), in addition to the initial 0.1.

We additionally assessed the association of DPP4 with REM sleep behavior disorder (RBD), one of prodromal symptoms of PD. We used the GWAS data of 1,061 RBD cases without other major neurological signs and 8,386 controls of European ancestry [25].

### MR analysis using eQTL results across different tissues and immune cell types

To assess the tissue-specific effects of genetically proxied DPP-4 inhibition on PD, we constructed genetic instruments using eQTL data from the Genotype-Tissue Expression (GTEx) consortium (v8 *cis*-eQTLs from 49 tissues) [26]. For each tissue, we selected independent SNPs (*r*^*2*^ <0.1) within ±200 ​kb of *DPP4* that were significantly associated with its expression (*p* ​< ​1 ​× ​10^−5^) and performed tissue-specific MR analyses for PD risk, overall and by sex.

Given the well-known immunomodulatory functions of DPP-4 (CD26), we further extended this analysis using immune cell types using eQTLs from the Database of Immune Cell Expression, eQTLs and Epigenomics (DICE) project [27]. For *DPP4*, two types of CD4^+^ T cells (memory T regulatory cells and TH2 cells) derived from blood samples were available.

### Mediation analysis with T2DM

To assess whether the effect of DPP-4Is on PD risk is mediated through the diabetes risk, we performed two-step MR analyses. First, we assessed the association between genetically proxied DPP-4Is and T2DM. Due to the limited SNP coverage in sex-stratified T2DM GWAS datasets – specifically, the absence of relevant IV_eQTL_ and IV_pQTL_ SNPs – this MR analysis was restricted to the combined-sex dataset. Next, we evaluated the impact of T2DM on PD risk using MR analysis, stratified by sex and overall. For T2DM, we utilized the GWAS summary statistics from the DIAMANTE consortium (80,154 cases and 853,816 controls) [28] for the combined dataset, and from the DIAGRAM consortium (men: 20,219 cases ​+ ​54,604 controls; women: 14,621 cases ​+ ​60,377 controls) [29], for sex-stratified analyses. Both datasets predominantly included individuals of European ancestry. Independent (r^2^ ​< ​0.001, *p*-value < 5 × 10^-8^) SNPs were selected as IVs.

In addition, we stratified the DPP-4 instruments based on their individual associations with T2DM risk. Specifically, we conducted single-SNP MR analyses using IV_eQTL_ and IV_pQTL_ separately, selecting SNPs associated (*p* ​≤ ​0.05) and not associated (*p* ​> ​0.05) with T2DM risk. We then estimated the causal effect of DPP-4 on PD risk within each stratum.

### Colocalization analysis

We performed colocalization analyses to assess whether genetic associations for DPP-4 expression or protein levels and PD risk share a common causal variant, using ‘coloc’ R package (v.6.0.0) [30]. It implements a Bayesian framework to estimate the posterior probabilities for five hypotheses: no association (H_0_), association with only the first trait (H_1_) or only the second trait (H_2_), both traits due to different causal variants (H_3_), and both traits due to a shared causal variant (H_4_). We calculated posterior probabilities (PP) for each hypothesis within the same window (±200 ​kb) in MR analyses. A high PP for H_4_ (PP_4_ ​> ​0.8) was interpreted as strong evidence for colocalization between QTL and PD association signals.

### Ethical approval

This study was exempt from Institutional Review Board (IRB) approval from Seoul National University (IRB No. E2105/003–010) since it solely used publicly available summary-level data from eQTL, pQTL, and GWAS data, none of which contain identifiable personal information. All studies used in this research obtained participant consent and ethical approval from their respective ethics review boards.

## Results

**Fig. 1:**
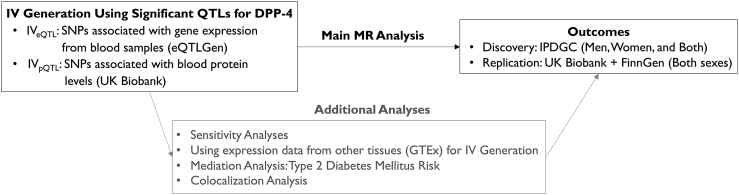
**Flowchart of the study.** For a target gene of DPP-4 inhibitors, IVs were generated using eQTL/pQTL results. Using an MR approach, we examined their impact on PD risk. Additional analyses included tissue-specific IV_eQTL_, sensitivity analyses, mediation analysis via T2DM, and colocalization analysis. Abbreviations: DPP-4, dipeptidyl peptidase-4; IV, instrumental variable; eQTL, expression quantitative trait loci; pQTL, protein QTL; MR, Mendelian randomization; PD, Parkinson's disease; IPDGC, International Parkinson's Disease Genomics Consortium; GTEx, Genotype-Tissue Expression.

Flowchart of this study is presented in Fig. 1. For *DPP4*, the target gene of DPP-4Is, we extracted 10 and 29 significant SNPs from eQTL and pQTL analyses, respectively. From the MR analyses of these instruments on the risk of PD, both IVs consistently increased the risk of PD, suggesting the drugs inhibiting DPP-4 may have a protective effect against PD. The effect sizes diminished in accordance with an increase in the biological distance between genetic variants and their targets: odds ratios (ORs) and 95% confidence intervals (CIs) using IV_eQTL_ and IV_pQTL_ were 1.78 [1.21–2.61], *p* ​= ​0.003 and 1.21 [1.02–1.45], *p* ​= ​0.032, respectively (Table 1). When stratified by sex, these increases were observed solely in men, exhibiting stronger associations and increased effect sizes: ORs (95% CIs) in men were 2.25 [1.55–3.28], *p* ​= ​2.1 × 10^-5^ and 1.35 [1.15–1.57], *p* ​= ​1.7 × 10^-4^, for IV_eQTL_ and IV_pQTL_. Analyses with different MR methods yielded consistent results in men, except for MR Egger method of IV_eQTL_ analysis. In women, the results were not significant across all IVs.

**Table 1:** MR analysis of genetically proxied DPP-4 inhibitors on the risk of Parkinson's disease (PD).

IV	Method	Men (n ​= ​12,054 ​+ ​11,999)^a^			Women (n ​= ​7,384 ​+ ​12,389)^a^			All (N ​= ​37,688 ​+ ​981,372)^a^		
		No. of SNPs	OR (95 ​% CI)	*P*-value	No. of SNPs	OR (95 ​% CI)	*P*-value	No. of SNPs	OR (95 ​% CI)	*P*-value
IV_eQTL_	IVW	9	2.25 (1.55–3.28)	**2.06E-05**	10	1.30 (0.88–1.92)	0.195	10	1.78 (1.21–2.61)	**0.003**
	Weighted Median		2.13 (1.32–3.43)	**0.002**		1.10 (0.66–1.83)	0.715		1.82 (1.12–2.97)	**0.016**
	Weighted Mode		2.16 (1.30–3.61)	**0.018**		1.04 (0.58–1.88)	0.889		1.68 (0.92–3.09)	0.127
	MR Egger		2.04 (0.88–4.74)	0.140		1.37 (0.60–3.13)	0.483		1.27 (0.56–2.88)	0.586
	MR-PRESSO		2.25 (1.61–3.16)	**0.001**		1.30 (0.98–1.71)	0.101		1.78 (1.21–2.61)	**0.016**
IV_pQTL_	IVW	25	1.35 (1.15–1.57)	**1.66E-04**	25	1.09 (0.92–1.29)	0.340	29	1.21 (1.02–1.45)	**0.032**
	Weighted Median		1.41 (1.13–1.76)	**0.002**		1.02 (0.80–1.29)	0.882		1.24 (0.98–1.57)	0.068
	Weighted Mode		1.42 (1.14–1.77)	**0.004**		1.06 (0.85–1.32)	0.614		1.25 (0.98–1.58)	0.082
	MR Egger		1.23 (0.88–1.74)	0.240		0.93 (0.64–1.34)	0.691		1.03 (0.69–1.54)	0.885
	MR-PRESSO		1.35 (1.16–1.56)	**5.52E-04**		1.09 (0.94–1.25)	0.262		1.21 (1.02–1.45)	**0.041**

Abbreviations: MR, Mendelian randomization; DPP-4, dipeptidyl peptidase-4; IV, instrumental variable; eQTL, expression quantitative trait loci; pQTL, protein QTL; OR, odds ratio; CI, confidence interval; IVW, inverse variance weighted. Bold indicates statistical significance at p < 0.05.

a Numbers of cases and controls, respectively.

As shown in the directed acyclic graph in Supplementary Fig. 1, the first (relevance) and third assumptions (exclusion restriction) are satisfied as *F* statistics (>10) and the results of pleiotropy test show (Supplementary Table 1). Plots for sensitivity analyses including results from diverse MR methods, single SNP and leave-one-out analysis (stratified for sex and overall) were presented in Supplementary Fig. 2-4 and 5-7, for IV_eQTL_, and IV_pQTL_, respectively. These analyses consistently supported significant associations between DPP-4 inhibition and overall PD, with the associations primarily driven by men. Results of the MR-Steiger directionality test indicated the direction of causal effects of DPP-4 on PD is true (*p* ​< ​0.001; Supplementary Table 2).

**Fig. 2:**
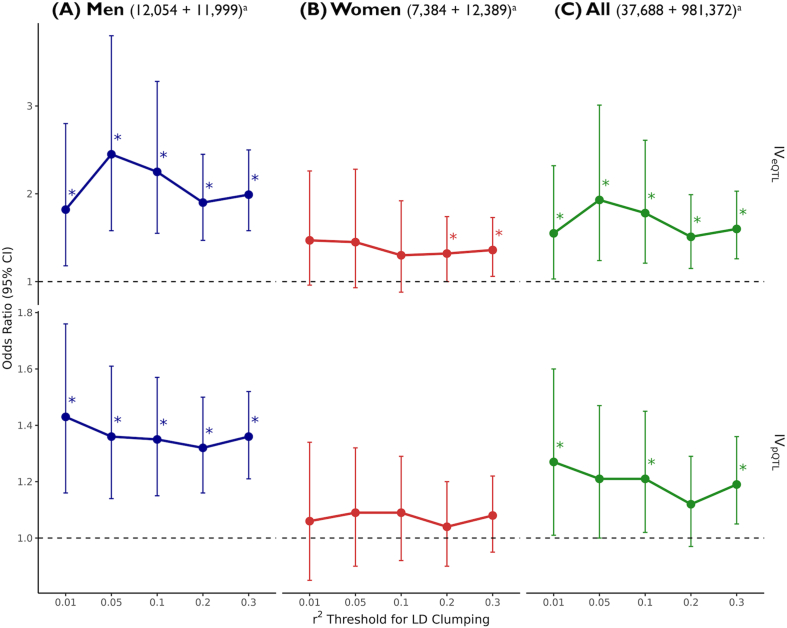
**Mendelian randomization (MR) analysis of DPP-4 inhibitors on Parkinson'sdisease (PD) risk across different SNP selection thresholds.** MR results assessing the effect of DPP-4 inhibition on PD risk in (A) men, (B) women, and (C) both sexes, using different r^2^ thresholds for SNP selection to construct the IV. Significant results (*p*-value ≤ 0.05) are marked with an asterisk. ^a^ The numbers of cases and controls are indicated in parentheses. Abbreviations: MR, Mendelian randomization; IV, instrumental variable; eQTL, expression quantitative trait loci; pQTL, protein quantitative trait loci; CI, confidence interval.

We also conducted the same MR analyses using different thresholds for clumping independent SNPs (r^2^ ​< ​0.3, 0.2, 0.05, and 0.01, in addition to 0.1) to generate instruments. In general, all IVs consistently presented an increase in PD risk with higher expression or protein levels of DPP-4 in men and in the combined dataset. In women, increased PD risk was observed only for IV_eQTL_ clumped using more liberal thresholds (r^2^ ​< ​0.3 and 0.2), while IV_pQTL_ showed no association with PD risk under any threshold (Fig. 2 and Supplementary Table 3).

In replication analyses using data from UK Biobank and FinnGen, the associations did not reach statistical significance under the default IVW methods but showed suggestive evidence of association (IV_eQTL_: OR 1.27 [0.99–1.61], p ​= ​0.055; IV_pQTL_: OR 1.1 [0.98–1.23], p ​= ​0.109). However, when alternative clumping thresholds were applied (r^2^ ​< ​0.3, 0.2, 0.05, or 0.01), and across different MR methods, statistically significant associations were observed in both IV_eQTL_ and IV_pQTL_ analyses (Supplementary Table 4). Additionally, we evaluated the effects of *DPP4* expression and its protein levels on RBD as a PD-related phenotype, and both IVs using showed positive or suggestive associations (IV_eQTL_: OR 4.17 [2.01–8.66], *p* ​= ​1.3 × 10^-4^; IV_pQTL_: 1.75 [1.18–2.59], *p* ​= ​0.005) (Supplementary Table 5).

From the GTEx data (49 tissues), we derived genetic instruments for DPP-4 inhibition, yielding seven significant *cis*-eQTLs across four tissues (Supplementary Table 6). Tissue-specific MR indicated increased PD risk for instruments in adrenal gland, thyroid, and transverse colon, as in the lung for males; no effects were detected in females. In immune cell analysis, higher *DPP4* expression in CD4^+^ T cells was associated with increased PD risk in males (OR ​= ​1.10, *p* ​= ​0.016), whereas the overall and female analyses showed no significant associations (Supplementary Table 7).

From the mediation analysis via T2DM, both IVs of DPP-4 showed suggestive associations with increased T2DM risk (IV_eQTL_: OR 1.14 [1.01–1.28], *p* ​= ​0.03; IV_pQTL_: 1.08 [1.03–1.14], *p* ​= ​0.004) (Supplementary Fig. 8). However, since no significant association was observed in any stratum of the MR analysis assessing the effect of T2DM on PD risk, we did not proceed with a two-step MR analysis. Instead, we stratified the DPP-4 instruments based on their individual association with T2DM risk using the single-SNP MR analysis, and subsequently evaluated their effects on PD. Among 8 and 28 SNPs comprising the IV_eQTL_ and IV_pQTL_, only two and five SNPs, respectively, were significantly associated with T2DM. In male participants, both T2DM-associated and non-associated IVs showed a trend toward increased risk of PD. In contrast, the MR analyses in women yielded no evidence of association between DPP-4 and PD risk, regardless of whether the instruments were associated with T2DM. In the combined dataset, the associations between DPP-4 and PD risk appeared to be primarily driven by SNPs not associated with T2DM (IV_eQTL_: OR 1.97 [1.12–3.48], *p* ​= ​0.019; IV_pQTL_: 1.37 (1.08–1.74), *p* ​= ​0.009), rather than by T2DM-associated SNPs (IV_eQTL_: OR 1.46 [0.67–3.19], *p* ​= ​0.34; IV_pQTL_: 1.15 [0.78–1.68], *p* ​= ​0.485) (Supplementary Table 8).

From the colocalization analysis using default prior probabilities, we did not observe strong evidence for a shared causal variant between gene expression or protein levels of DPP-4 and PD risk (Supplementary Table 9). While both the eQTL and pQTL datasets suggested a weak signal of shared causal variants in men (PP_4_ ​= ​0.403 and 0.402, respectively), the results in women or the combined dataset showed high posterior probabilities for H_1_ (QTL).

## Discussion

This study suggests that DPP-4Is could be effective in preventing PD in men, supporting their potential prioritization for male diabetic patients at higher risk of PD. Our genetic proxies of DPP-4, derived from both gene expression and protein data, consistently showed significant associations. Additionally, our findings revealed sex differences suggesting distinct relationships between DPP-4 and PD.

To date, no randomized controlled trials have specifically investigated DPP-4Is for PD treatment, highlighting the importance of our drug-target MR approach in exploring this potential therapeutic avenue. Drug-target MR has been proposed to explore repurposing opportunities of existing drugs for other diseases, especially where clinical trials may not be feasible. One of the major caveats of this approach, however, is the limitation of genetic proxies to accurately represent the effects of the drug. To address this concern, we constructed genetic IVs leveraging proteomics and gene-expression profiles (data used: eQTL and pQTL; r^2^ threshold for selecting genetic instruments ​= ​0.3, 0.2, 0.1, 0.05, and 0.01). These genetic proxies were validated for their effects on target phenotype, T2DM. The multi-omics-based drug-target MR approach used in this study offers advantages over traditional genetic IV approaches by providing a more comprehensive representation of drug effects, incorporating both gene expression and protein level data.

Our main MR analysis revealed a potential protective effect of DPP-4 inhibition on male PD, with consistent results across various MR methods. Moreover, these effects reduced both risk of PD and RBD, reinforcing the protective effect of DPP-4Is on male PD. Conversely, no effect of DPP-4 inhibition was observed on female PD. Given that PD demonstrates sex differences in its epidemiological and clinical features, with hormonal and neuroendocrine factors proposed as underlying mechanisms [31], our MR analyses across different tissues showed positive associations with overall PD when using IVs of *DPP-4* expression in the adrenal gland and thyroid, possibly implicating the endocrine system in these sex differences. Moreover, there are reports that DPP-4Is have beneficial effects only in male T2DM patients, such as reducing carotid artery intima-media thickness [32] or being associated with a decreased risk of cardiovascular outcomes [33] compared to placebo or other conventional therapies, further supporting the sex-specific effects of DPP-4 inhibition.

We found that higher *DPP4* expression in the transverse colon was significantly associated with overall PD risk, reinforcing a gut-brain axis contribution in PD pathogenesis. Converging evidence supports an inflammatory mechanism: Jeong et al. demonstrated that DPP-4 inhibition reduces colonic inflammation and downstream α-synuclein pathology in rotenone-treated PD mouse models [34], and our immune-cell MR analysis showed that higher CD4^+^ T cell *DPP4* expression was associated with increased PD risk in males. Collectively, these data suggest that DPP-4 inhibition may be protective in PD through immune-inflammatory modulation.

Another possible explanation for the observed sex-specific effects of DPP-4 inhibition on PD risk could be the underlying phenotypic differences between males and females in PD manifestation. This hypothesis aligns with the growing recognition that PD represents a set of heterogeneous conditions rather than a single disease entity, prompting efforts to subclassify the disease. One classification method that implies differences in early pathogenesis is based on the presence of premotor RBD. Patients with premotor RBD tend to have later onset, severe autonomic dysfunction, symmetric involvement, and more cognitive decline [35]. Intriguingly, previous studies on patients with isolated RBD have consistently included more males than females including the RBD GWAS used in this study (81 ​% male) [25], suggesting males are more likely to exhibit this distinct phenotype. In our study, RBD, contrary to PD, was strongly associated with *DPP-4* expression even without sex-stratification. This finding may reflect the phenotypic differences between males and females in PD and RBD manifestation, warranting further investigation.

We also performed a mediation analysis to assess whether the effect of DPP-4 on PD risk is mediated through T2DM. While DPP-4 showed a suggestive association with T2DM as expected, we found no evidence that T2DM itself increases PD risk, consistent with previous MR report [36], and the main association between DPP-4 and PD was primarily driven by SNPs not associated with T2DM. These findings suggest that the observed DPP-4 and PD link is independent of T2DM. We further explored whether this association might be explained by altered GLP-1 signaling. However, our GLP1R-based drug-target MR showed no significant association with PD risk. This may reflect that receptor expression or protein abundance does not necessarily correspond to receptor activation status [37], as also observed in our analysis showing a null association with T2DM (data not shown).

Although the mechanisms underlying the sex-specific effects of DPP-4 inhibition on PD risk remains unclear, our findings suggest that DPP-4Is may be effective in preventing PD in men. The robustness of this association was supported by diverse IVs (using both transcriptomic and proteomic data, and eQTL data from various tissues and different thresholds for IV selection) and sensitivity analyses (replication analysis and RBD as a PD-related phenotype, and MR analyses using different methods). No significant colocalization was observed between DPP-4 gene expression or protein levels and PD risk under default prior assumptions. However, after relaxing the priors to account for the limited overlap between QTLs and GWAS signals (p_1_ ​= ​1 ​× ​10^−4^, p_2_ ​= ​1 ​× ​10^−3^, p_12_ ​= ​1 ​× ​10^−4^), strong evidence for colocalization emerged specifically in men, for both the eQTL and pQTL datasets (PP_4_ ​= ​0.819 and 0.816, respectively). In contrast, no meaningful colocalization was detected in women or the combined dataset under the same settings. These findings suggest that potential sex-specific genetic mechanisms may underlie the observed associations.

Our study has several limitations. First, while we propose a potential effect of DPP-4Is on PD and its related traits, the scope of this study does not include the mechanism of this potential repurposing. Additionally, a drug-target MR study cannot fully replicate clinical trials, as it mimics low-dose lifelong exposures rather than reflecting high-dose short-term drug use in trials. Notably, the effect sizes from our analysis are estimated per unit increase of normalized gene expression or protein levels and do not reflect the actual dose of DPP-4Is. Genetic instruments using specific target proteins of drugs may not account for complex network between other proteins. Third, we used eQTL and pQTL data generated from sex-combined analyses, which may not fully capture sex-specific genetic effects. However, in our separate MR analyses using sex-stratified pQTL results with the UK Biobank dataset, the results (men: OR 2.47 [1.47–4.13], *p* ​= ​6.2e-4; women: OR 1.04 [0.54–1.98], *p* ​= ​0.912) were largely consistent with those derived from the sex-combined data, suggesting that the observed associations are unlikely to be driven solely by sex-related differences in genetic regulation. Fourth, our use of DPP-4 expression and protein levels as instruments may not fully capture the functional effects of DPP-4 inhibition. To address this, we conducted additional analyses using type 2 diabetes as a functional proxy, which suggested that the association with PD is unlikely to be mediated through glycemic pathways. Fifth, the absence of significant associations in women may reflect insufficient statistical power and should therefore be interpreted with caution. This could be due to the smaller sample size in female datasets, as well as the relatively weaker effect sizes observed in women. Finally, our results are limited to the European population and not fully validated in non-European populations. Caution should be taken when generalizing these findings to other populations.

In conclusion, this study highlights the protective role of DPP-4Is in male PD using genetic instruments for this drug target. Notably, the sex differences observed indicate a varying impact of DPP-4Is between males and females. Our findings support the potential of DPP-4Is as a promising therapeutic option in the prevention of PD in men.

## Author contributions

**Joo-Yeon Lee:** Conceived and designed the analysis, Collected the data, Contributed data or analysis tools, Performed the analysis, Wrote the paper.

**Don Gueu Park:** Conceived and designed the analysis, Wrote the paper.

**Joohon Sung:** Conceived and designed the analysis, Wrote the paper.

## Declaration of competing interest

The authors declare the following financial interests/personal relationships which may be considered as potential competing interests: Joo-Yeon Lee reports financial support was provided by National Research Foundation of Korea. Don Gueu Park reports financial support was provided by National Research Foundation of Korea. Joohon Sung reports financial support was provided by Ministry of Food and Drug Safety. If there are other authors, they declare that they have no known competing financial interests or personal relationships that could have appeared to influence the work reported in this paper.
