# Supplementary material for: Repurposing dipeptidyl peptidase-4 inhibitor for Parkinson's disease prevention: A drug-target Mendelian randomization study
Source: Neurotherapeutics. 2025 Dec 29;23(1):e00815. doi: 10.1016/j.neurot.2025.e00815 (PMC12976525; doi:10.1016/j.neurot.2025.e00815)
Supplement: Multimedia component 1 [file mmc1.docx]

**Supplementary Material**

**List of Figures**

Supplementary Figure 1. Directed acyclic graph (DAG) for the MR Analysis of DPP-4 Inhibitors on the Parkinson’s Disease (PD) Risk.

Supplementary Figure 2. Scatter Plots for the Different MR Methods of *DPP4* expression on the PD Risk in (A) Men, (B) Women, and (C) All Participants.

Supplementary Figure 3. Forest Plots for the Single SNP MR Analysis of *DPP4* expression on the PD Risk in (A) Men, (B) Women, and (C) All Participants.

Supplementary Figure 4. Forest Plots for the Leave-One-Out Analysis of *DPP4* expression on the PD Risk in (A) Men, (B) Women, and (C) All Participants.

Supplementary Figure 5. Scatter Plots for the Different MR Methods of DPP-4 Proteins levels on the PD Risk in (A) Men, (B) Women, and (C) All Participants.

Supplementary Figure 6. Forest Plots for the Single SNP MR Analysis of DPP-4 Proteins levels on the PD Risk in (A) Men, (B) Women, and (C) All Participants.

Supplementary Figure 7. Forest Plots for the Leave-One-Out Analysis of DPP-4 Proteins levels on the PD Risk in (A) Men, (B) Women, and (C) All Participants.

Supplementary Figure 8. Graphical Summary of Mediation and Colocalization Analyses Supporting a Direct Role of DPP-4 in PD.

**List of Tables**

Supplementary Table 1. Statistical Validation of MR Analyses

Supplementary Table 2. Results from the MR-Steiger Directionality Test

Supplementary Table 3. MR Analysis of Genetically Proxied DPP-4 Inhibitors on the Risk of PD After Excluding the UK Biobank Participants

Supplementary Table 4. MR Analysis of Genetically Proxied DPP-4 Inhibitors on the Risk of PD Using Meta-Analyses of FinnGen and UK Biobank (Replication Phase)

Supplementary Table 5. MR Analysis of Genetically Proxied DPP-4 Inhibitors on the Risk of REM Sleep Behavior Disorder (RBD)

Supplementary Table 6. MR Analysis of Genetically Proxied DPP-4 Inhibitors Using Gene Expression Data Across Various Tissues on the Risk of PD

Supplementary Table 7. MR Analysis of Genetically Proxied DPP-4 Inhibitors Using Gene Expression Data in CD4+ T cells on the Risk of PD

Supplementary Table 8. Mediation MR of Genetically Proxied DPP-4 Inhibition on the PD Risk via Type 2 Diabetes

Supplementary Table 9. Colocalization Analyses between Expression/Protein Quantitative Trait Loci and PD Risk

**STROBE-MR Checklist**


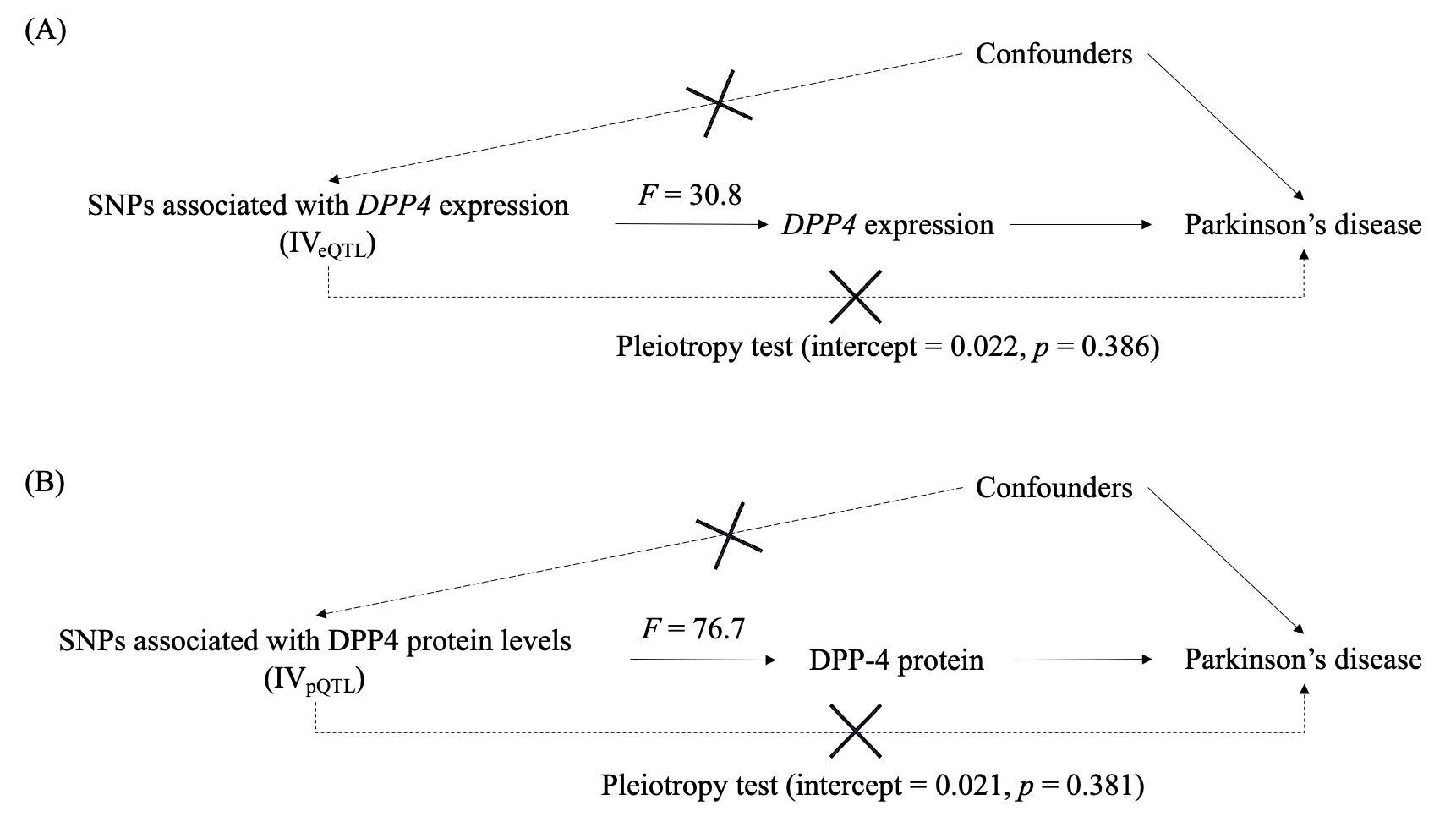


**Supplementary Figure 1. Directed acyclic graph (DAG) for the MR Analysis of DPP-4 Inhibitors on the Parkinson’s Disease (PD) Risk.** Three fundamental assumptions behind the MR analysis using (A) IV_eQTL_ and (B) IV*_cis_*_-pQTL_ are shown. Relevance assumption and exclusion restriction assumptions were assessed with *F* statistics (all *F* > 10) and pleiotropy tests (all *p*-values for Egger intercept > 0.05). Independence assumption could not be evaluated with GWAS summary data; however, it is unlikely that the IVs for specific drug targets would be affected by common confounders.


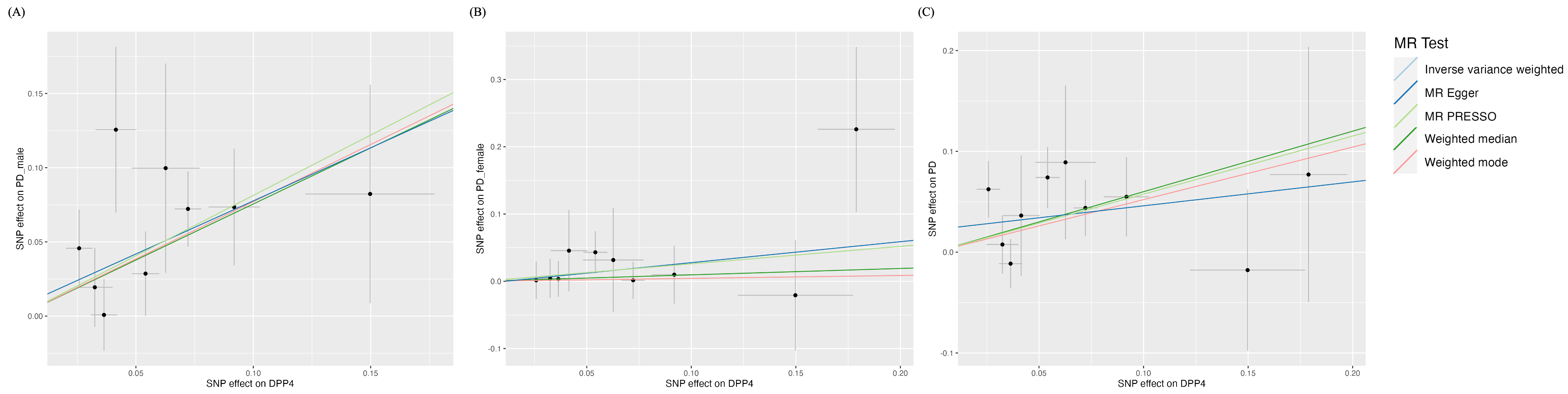


**Supplementary Figure 2. Scatter Plots for the Different MR Methods of *DPP4* Expression on the PD Risk in (A) Men, (B) Women, and (C) All Participants.**


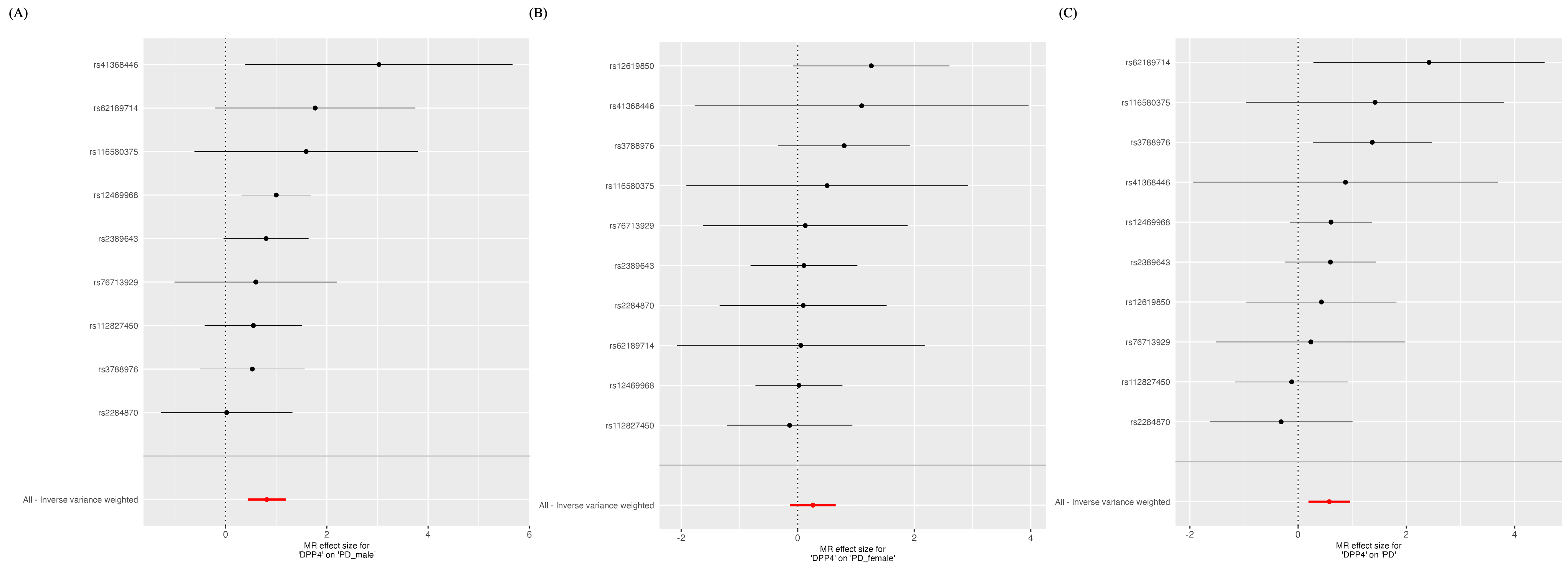


**Supplementary Figure 3. Forest Plots for the Single SNP MR Analysis of *DPP4* Expression on the PD Risk in (A) Men, (B) Women, and (C) All Participants.**

**
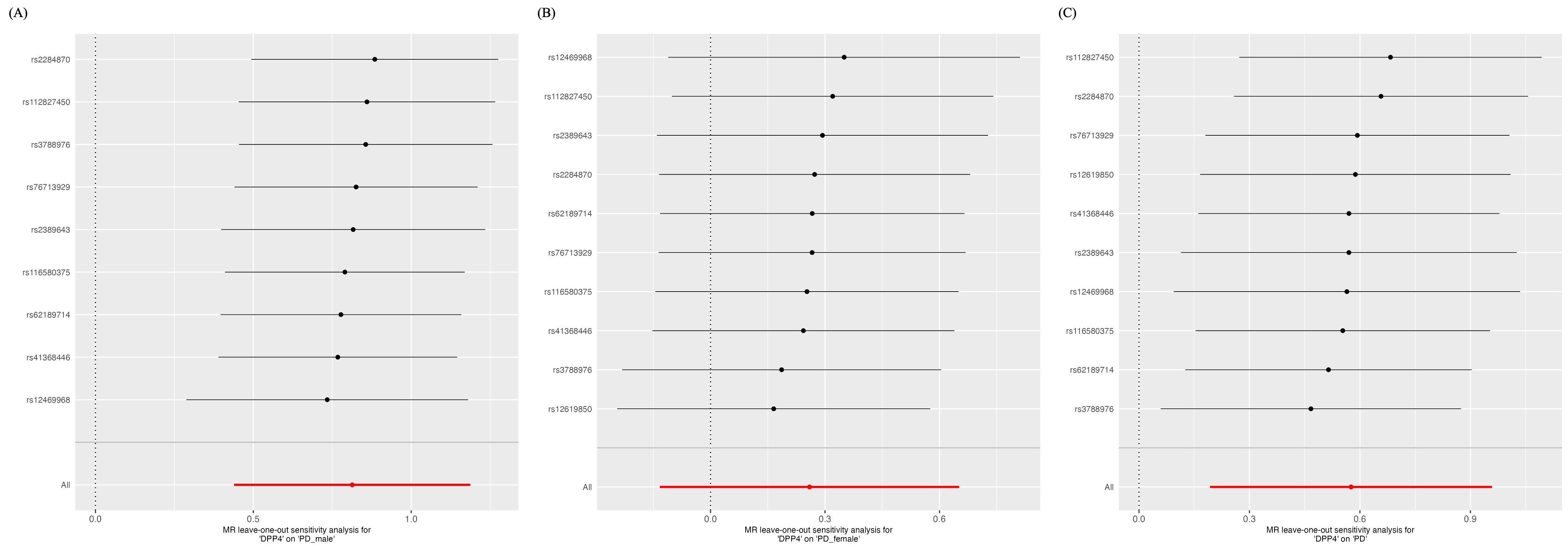
**

**Supplementary Figure 4. Forest Plots for the Leave-One-Out Analysis of *DPP4* Expression on the PD Risk in (A) Men, (B) Women, and (C) All Participants.**


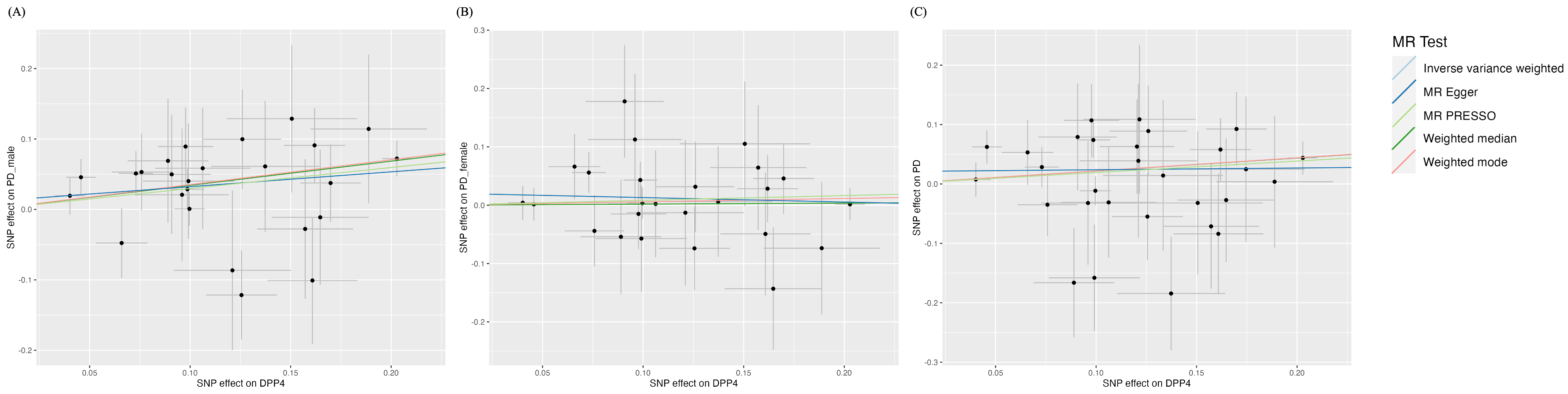


**Supplementary Figure 5. Scatter Plots for the Different MR Methods of DPP-4 Protein Levels on the PD Risk in (A) Men, (B) Women, and (C) All Participants.**


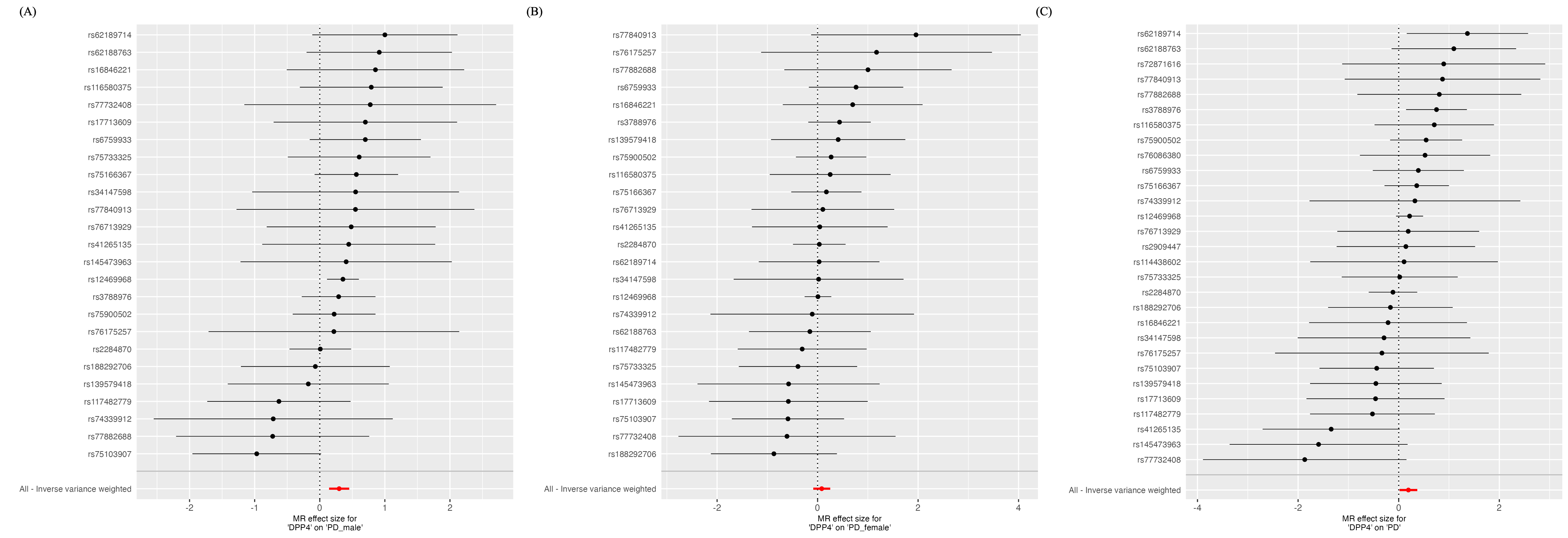


**Supplementary Figure 6. Forest Plots for the Single SNP MR Analysis of DPP-4 Protein Levels on the PD Risk in (A) Men, (B) Women, and (C) All Participants.**


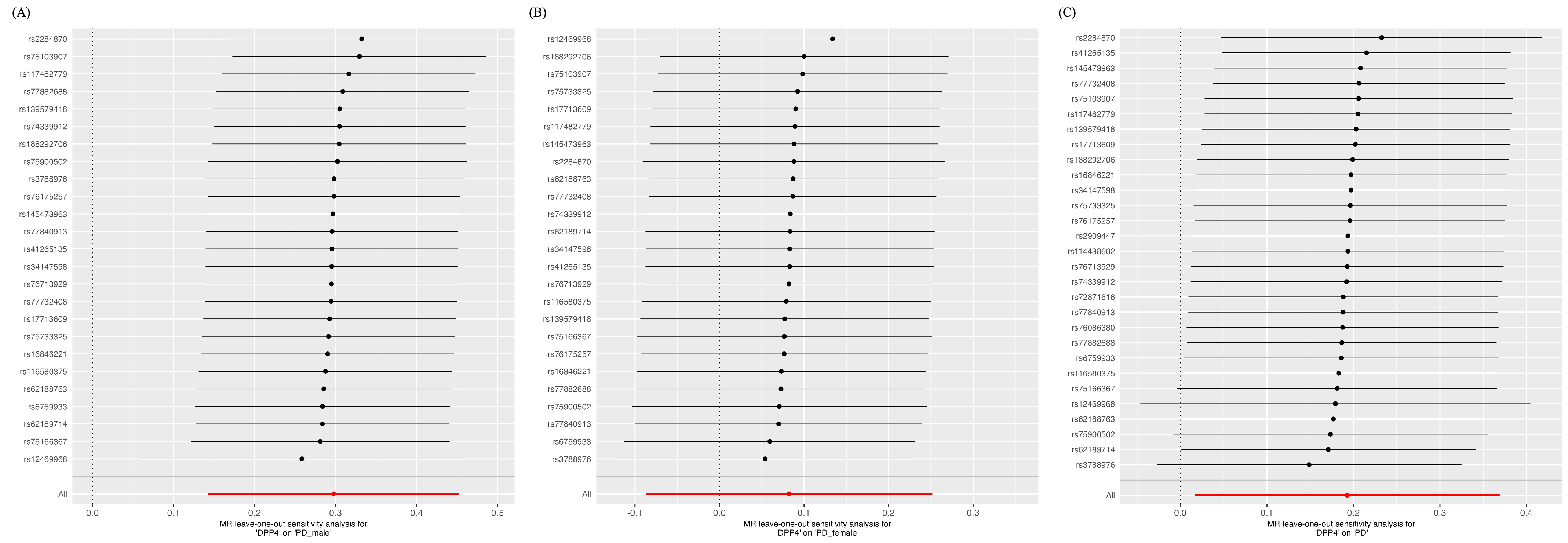


**Supplementary Figure 7. Forest Plots for the Leave-One-Out Analysis of DPP-4 Protein Levels on the PD Risk in (A) Men, (B) Women, and (C) All Participants.**

**
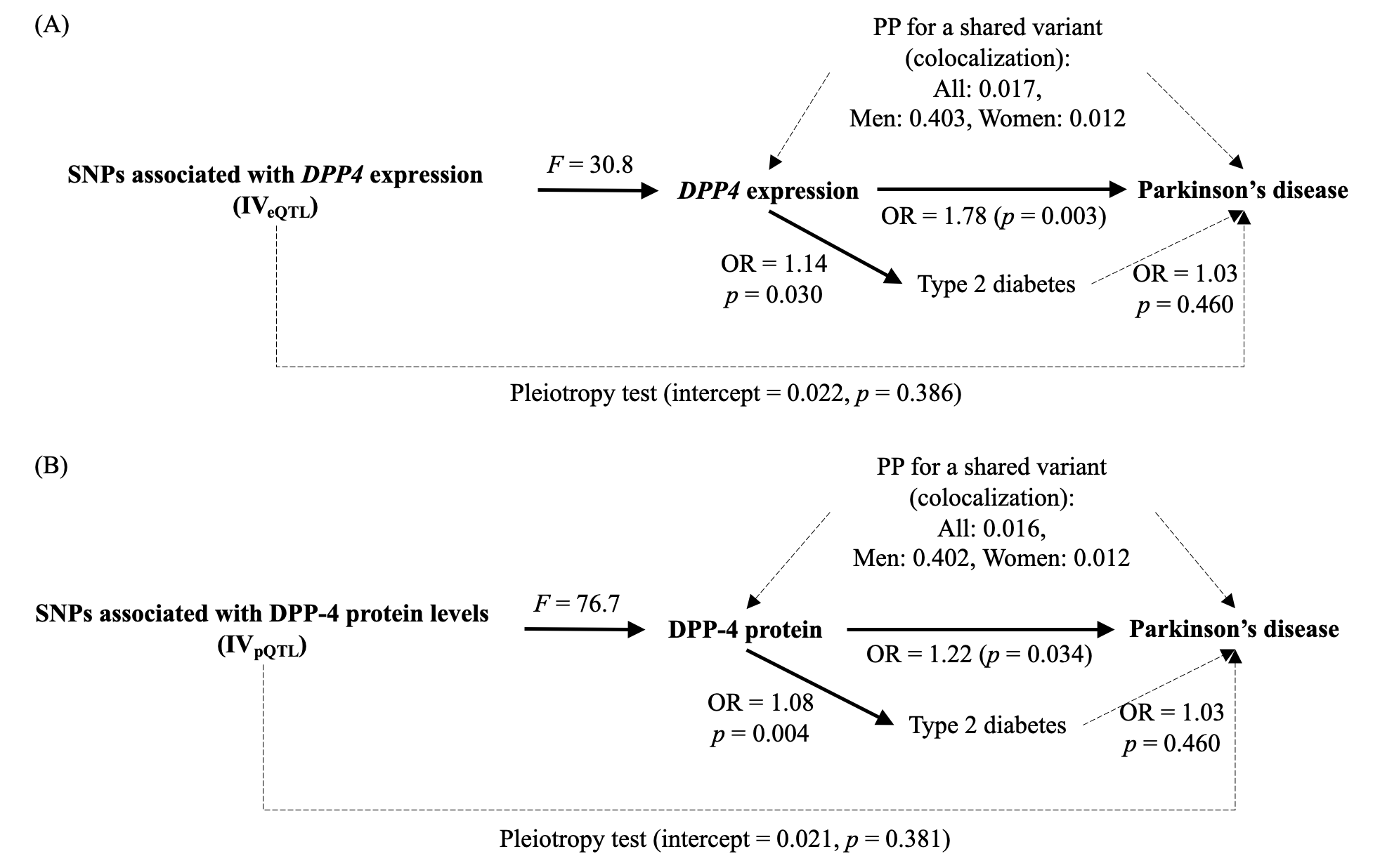
**

**Supplementary Figure 8. Graphical Summary of Mediation and Colocalization Analyses Supporting a Direct Role of DPP-4 in PD.** This diagram illustrates the potential pathways through which DPP-4 expression/protein levels may influence PD risk, using (A) IV_eQTL_, and (B) IV_pQTL_. While DPP-4 instruments are associated with type 2 diabetes, our mediation analysis showed no causal effect of diabetes on PD, suggesting that DPP-4 may act via an independent mechanism. Colocalization analysis revealed evidence of a shared causal variant between DPP-4 protein levels and PD risk in men only: PP (Posterior probability) for a shared variant was 0.403 and 0.402 for male PD risk and DPP-4 expression, or protein, respectively. Solid lines represent relationships supported by empirical evidence, whereas dashed lines indicate pathways that were not supported by the data.

**Supplementary Table 1. Statistical Validation of MR Analyses**

| **IV** | **Sex** | **Heterogeneity** | | **Pleiotropy (MR-Egger Analysis)** | | | ***I^2^_GX_* statistic** | ***F* statistic** | **Power** |
| --- | --- | --- | --- | --- | --- | --- | --- | --- | --- |
|  |  | **Q-statistics** | ***P*-value** | **Intercept** | **S.E.** | ***P*-value** |  |  |  |
| IV_eQTL_ | Men | 6.454 | 0.596 | 0.006 | 0.024 | 0.806 | 0.980 | 27.522 | 1 |
|  | Women | 4.511 | 0.875 | -0.003 | 0.025 | 0.892 | 0.981 | 30.762 | 0.72 |
|  | All | 9.024 | 0.435 | 0.022 | 0.024 | 0.386 | 0.981 | 30.762 | 1 |
| IV_pQTL_ | Men | 21.465 | 0.611 | 0.011 | 0.020 | 0.580 | 0.988 | 83.649 | 1 |
|  | Women | 16.516 | 0.869 | 0.021 | 0.022 | 0.357 | 0.988 | 83.649 | 0.28 |
|  | All | 32.643 | 0.249 | 0.021 | 0.024 | 0.381 | 0.987 | 76.669 | 1 |

I^2^_GX_​ statistics was calculated using the method proposed by Bowden et al. (2016), based on Cochran’s Q for SNP–exposure estimates:

${I^{2}}_{GX}=\frac{Q-(L-1)}{Q}$, where $Q=\sum_{j=1}^{L} \frac{\left( \hat{\gamma}_{j}-\bar{\hat{\gamma}} \right)^{2}}{{\sigma^{2}}_{X_{j}}}$. Here, *L* denotes the total number of instrumental variables, $\hat{\gamma}_{j}$ is the SNP–exposure association estimate for variant *j*, ${\sigma^{2}}_{X_{j}}$ is its corresponding variance, and $\bar{\hat{\gamma}}$ is the inverse-variance weighted mean of all $\hat{\gamma}_{j}.$

Abbreviations: MR, Mendelian randomization; SE: standard error; IV, instrumental variable; eQTL, expression quantitative trait loci; pQTL, protein QTL.

**Supplementary Table 2. Results from the MR-Steiger Directionality Test**

| **IV** | **Sex** | **r^2^.exposure** | **r2.outcome** | **Correct causal direction** | ***P*-value** |
| --- | --- | --- | --- | --- | --- |
| IV_eQTL_ | Men | 1.55% | 0.10% | TRUE | <0.001 |
|  | Women | 1.93% | 0.03% | TRUE | <0.001 |
|  | All | 1.93% | 0.00% | TRUE | <0.001 |
| IV_pQTL_ | Men | 5.99% | 0.15% | TRUE | <0.001 |
|  | Women | 5.99% | 0.09% | TRUE | <0.001 |
|  | All | 6.34% | 0.00% | TRUE | <0.001 |

Abbreviations: MR, Mendelian randomization; IV, instrumental variable; eQTL, expression quantitative trait loci; pQTL, protein QTL.

**Supplementary Table 3. MR Analysis of Genetically Proxied DPP-4 Inhibitors on the PD Risk Using Different Thresholds for IV Selection**

| **IV** | **R^2^ Threshold for IV Selection** | **Men (n=12,054 + 11,999)^*^** | | | **Women (n=7,384 + 12,389)^*^** | | | **All (N=37,688 + 981,372)^*^** | | |
| --- | --- | --- | --- | --- | --- | --- | --- | --- | --- | --- |
|  |  | **No. of SNPs** | **OR (95% CI)** | ***P*-value** | **No. of SNPs** | **OR (95% CI)** | ***P*-value** | **No. of SNPs** | **OR (95% CI)** | ***P*-value** |
| IV_eQTL_ | 0.3 | 18 | 1.99 (1.58-2.5) | **3.75E-09** | 19 | 1.36 (1.06-1.73) | **0.015** | 19 | 1.6 (1.26-2.03) | **9.37E-05** |
|  | 0.2 | 14 | 1.9 (1.47-2.45) | **1.04.E-06** | 15 | 1.32 (1-1.74) | **0.048** | 15 | 1.51 (1.15-1.99) | **0.003** |
|  | 0.1 | 9 | 2.25 (1.55-3.28) | **2.06.E-05** | 10 | 1.3 (0.88-1.92) | 0.195 | 10 | 1.78 (1.21-2.61) | **0.003** |
|  | 0.05 | 6 | 2.45 (1.58-3.8) | **6.03.E-05** | 7 | 1.45 (0.93-2.28) | 0.103 | 7 | 1.93 (1.24-3.01) | **0.004** |
|  | 0.01 | 3 | 1.82 (1.18-2.8) | **0.007** | 4 | 1.47 (0.96-2.26) | 0.074 | 4 | 1.55 (1.03-2.32) | **0.035** |
| IV_pQTL_ | 0.3 | 41 | 1.36 (1.21-1.52) | **1.E-07** | 41 | 1.08 (0.95-1.22) | 0.221 | 46 | 1.19 (1.05-1.36) | **0.008** |
|  | 0.2 | 32 | 1.32 (1.16-1.5) | **3.E-05** | 32 | 1.04 (0.9-1.2) | 0.596 | 37 | 1.12 (0.97-1.29) | 0.109 |
|  | 0.1 | 25 | 1.35 (1.15-1.57) | **1.66E-04** | 25 | 1.09 (0.92-1.29) | 0.34 | 29 | 1.21 (1.02-1.45) | **0.032** |
|  | 0.05 | 19 | 1.36 (1.14-1.61) | **0.001** | 19 | 1.09 (0.9-1.32) | 0.367 | 23 | 1.21 (1-1.47) | 0.051 |
|  | 0.01 | 9 | 1.43 (1.16-1.76) | **9.16E-04** | 9 | 1.06 (0.85-1.34) | 0.595 | 10 | 1.27 (1.01-1.6) | **0.039** |

^*^ Numbers of cases and controls, respectively.

Abbreviations: MR, Mendelian randomization; DPP-4, dipeptidyl peptidase-4; PD: Parkinson’s disease; IV: instrumental variable, eQTL: expression quantitative trait loci, pQTL: protein QTL, LD, linkage disequilibrium; OR, odds ratio; CI, confidence interval.

**Supplementary Table 4. MR Analysis of Genetically Proxied DPP-4 Inhibitors on the Risk of the PD Risk Using Meta-Analyses of FinnGen and UK Biobank (Replication Phase) ^a^**

| **IV** | **Method** | **R^2^ < 0.3** | | **R^2^ < 0.2** | | **R^2^ < 0.1** | | **R^2^ < 0.05** | | **R^2^ < 0.01** | |
| --- | --- | --- | --- | --- | --- | --- | --- | --- | --- | --- | --- |
|  |  | **OR (95% CI)** | ***P*** | **OR (95% CI)** | ***P*** | **OR (95% CI)** | ***P*** | **OR (95% CI)** | ***P*** | **OR (95% CI)** | ***P*** |
| IV_eQTL_ | IVW | 1.18 (1.02-1.37) | **0.028** | 1.16 (0.98-1.37) | 0.086 | 1.27 (0.99-1.61) | 0.055 | 1.39 (1.07-1.82) | **0.015** | 1.17 (0.9-1.51) | 0.231 |
|  | Weighted Median | 1.22 (0.99-1.5) | 0.059 | 1.1 (0.86-1.4) | 0.446 | 1.49 (1.08-2.08) | **0.017** | 1.51 (1.08-2.12) | **0.015** | 1.13 (0.82-1.55) | 0.448 |
|  | Weighted Mode | 1.34 (0.98-1.84) | 0.087 | 1.03 (0.76-1.39) | 0.869 | 1.54 (1.06-2.24) | 0.051 | 1.55 (1.09-2.19) | **0.049** | 1 (0.68-1.48) | 0.983 |
|  | MR Egger | 1.06 (0.77-1.46) | 0.707 | 1.05 (0.73-1.5) | 0.811 | 1.41 (0.81-2.44) | 0.257 | 2.29 (0.8-6.55) | 0.182 | 0.88 (0.49-1.58) | 0.706 |
|  | MR-PRESSO | 1.18 (1.04-1.34) | 0.019 | 1.16 (1.01-1.33) | 0.061 | 1.27 (1-1.6) | 0.081 | 1.39 (1.11-1.75) | **0.029** | 1.17 (0.94-1.45) | 0.25 |
| IV_pQTL_ | IVW | 1.09 (1.02-1.17) | **0.018** | 1.07 (0.98-1.16) | 0.118 | 1.1 (0.98-1.23) | 0.109 | 1.08 (0.97-1.2) | 0.17 | 1.16 (1-1.33) | **0.045** |
|  | Weighted Median | 1.15 (1.03-1.27) | **0.011** | 1.09 (0.97-1.24) | 0.147 | 1.15 (0.99-1.33) | 0.06 | 1.14 (0.99-1.31) | 0.07 | 1.16 (0.99-1.35) | 0.067 |
|  | Weighted Mode | 1.13 (1.01-1.27) | **0.044** | 1.1 (0.98-1.24) | 0.116 | 1.16 (1-1.35) | 0.055 | 1.14 (0.99-1.31) | 0.09 | 1.17 (0.99-1.37) | 0.093 |
|  | MR Egger | 1.13 (0.96-1.34) | 0.154 | 1.18 (0.96-1.45) | 0.122 | 1.12 (0.87-1.44) | 0.402 | 1.13 (0.88-1.46) | 0.356 | 1.14 (0.8-1.62) | 0.488 |
|  | MR-PRESSO | 1.09 (1.02-1.17) | **0.012** | 1.07 (0.99-1.16) | 0.118 | 1.1 (0.98-1.23) | 0.121 | 1.08 (0.98-1.18) | 0.126 | 1.16 (1.01-1.32) | 0.066 |

^a^ Numbers of samples are 7,521 cases and 897,736 controls.

Abbreviations: MR, Mendelian randomization; DPP-4, dipeptidyl peptidase-4; PD: Parkinson’s disease; IV, instrumental variable; eQTL, expression quantitative trait loci; pQTL, protein QTL; OR, odds ratio; CI, confidence interval; IVW, inverse variance weighted.

**Supplementary Table 5. MR Analysis of Genetically Proxied DPP-4 Inhibitors on the Risk of REM Sleep Behavior Disorder (RBD)**

| **IV** | **Method** | **R^2^ < 0.3** | | **R^2^ < 0.2** | | **R^2^ < 0.1** | | **R^2^ < 0.05** | | **R^2^ < 0.01** | |
| --- | --- | --- | --- | --- | --- | --- | --- | --- | --- | --- | --- |
|  |  | **OR (95% CI)** | ***P*** | **OR (95% CI)** | ***P*** | **OR (95% CI)** | ***P*** | **OR (95% CI)** | ***P*** | **OR (95% CI)** | ***P*** |
| IV_eQTL_ | IVW | 3.14 (1.77-5.56) | **8.8.E-05** | 3.27 (1.94-5.5) | **8.7.E-06** | 4.17 (2.01-8.66) | **1.3.E-04** | 3.54 (1.53-8.21) | **0.003** | 2.18 (0.95-5.01) | 0.067 |
|  | Weighted Median | 2.62 (1.29-5.3) | **0.008** | 2.87 (1.35-6.08) | **0.006** | 2.72 (1.11-6.68) | **0.029** | 2.72 (0.96-7.71) | 0.06 | 2.65 (0.97-7.24) | 0.057 |
|  | Weighted Mode | 1.79 (0.51-6.23) | 0.375 | 3.57 (1.26-10.15) | **0.032** | 2.75 (0.98-7.75) | 0.088 | 3.03 (0.94-9.78) | 0.114 | 2.97 (0.77-11.52) | 0.214 |
|  | MR Egger | 1.6 (0.46-5.57) | 0.47 | 1.62 (0.51-5.14) | 0.429 | 2.09 (0.46-9.64) | 0.370 | 1.6 (0.1-26.52) | 0.757 | 0.74 (0.1-5.5) | 0.797 |
|  | MR-PRESSO | 3.14 (1.77-5.56) | **0.001** | 3.27 (1.95-5.47) | **5.1.E-04** | 4.17 (2.35-7.4) | **8.6.E-04** | 3.54 (1.68-7.46) | **0.016** | 2.18 (1.08-4.39) | 0.118 |
| IV_pQTL_ | IVW | 1.9 (1.42-2.54) | **1.7.E-05** | 2.07 (1.49-2.87) | **1.4.E-05** | 1.75 (1.18-2.59) | **5.0.E-03** | 1.68 (1.14-2.5) | **0.009** | 1.81 (1.18-2.79) | **0.006** |
|  | Weighted Median | 1.6 (1.1-2.34) | **0.015** | 2.19 (1.47-3.27) | **1.2.E-04** | 1.45 (0.91-2.3) | 0.119 | 1.49 (0.94-2.36) | 0.086 | 1.56 (0.96-2.53) | 0.071 |
|  | Weighted Mode | 1.69 (1.14-2.5) | **0.012** | 1.82 (1.24-2.69) | **0.004** | 1.38 (0.87-2.2) | 0.185 | 1.48 (0.95-2.29) | 0.096 | 1.43 (0.88-2.31) | 0.183 |
|  | MR Egger | 1.51 (0.78-2.92) | 0.226 | 1.29 (0.59-2.82) | 0.53 | 1.07 (0.47-2.43) | 0.864 | 1.06 (0.44-2.57) | 0.892 | 0.55 (0.19-1.58) | 0.301 |
|  | MR-PRESSO | 1.9 (1.42-2.54) | **9.0.E-05** | 2.07 (1.49-2.87) | **1.1.E-04** | 1.75 (1.18-2.59) | **9.0.E-03** | 1.68 (1.14-2.5) | **0.017** | 1.81 (1.19-2.77) | **0.023** |

^a^ Numbers of samples are 1,061 cases and 8,386 controls.

Abbreviations: MR, Mendelian randomization; REM, rapid eye movement; DPP-4, dipeptidyl peptidase-4; IV: instrumental variable; eQTL, expression quantitative trait loci; pQTL, protein QTL; IVW, inverse variance weighted; OR, odds ratio; CI, confidence interval.

**Supplementary Table 6. MR Analysis of Genetically Proxied DPP-4 Inhibitors Using Gene Expression Data Across Various Tissues on the Risk of PD**

| **Tissue** | **#SNPs** | **Men (n=12,054 + 11,999)^*^** | | **Women (n=7,384 + 12,389)^*^** | | **All (N=37,688 + 981,372)^*^** | |
| --- | --- | --- | --- | --- | --- | --- | --- |
|  |  | **OR (95% CI)** | ***P*-value** | **OR (95% CI)** | ***P*-value** | **OR (95% CI)** | ***P*-value** |
| Adrenal gland | 1 | 1.06 (0.97-1.14) | 0.227 | 0.99 (0.89-1.08) | 0.761 | 1.12 (1.02-1.21) | **0.024** |
| Lung | 4 | 1.35 (1.2-1.49) | **8.73E-05** | 1.06 (0.9-1.22) | 0.476 | 1.15 (0.99-1.3) | 0.085 |
| Thyroid | 1 | 1.24 (0.94-1.53) | 0.156 | 0.98 (0.67-1.3) | 0.924 | 1.44 (1.12-1.76) | **0.025** |
| Transverse colon | 1 | 0.93 (0.77-1.08) | 0.313 | 1.14 (0.98-1.31) | 0.115 | 1.21 (1.06-1.37) | **0.014** |

^*^ Numbers of cases and controls, respectively.

Abbreviations: MR, Mendelian randomization; DPP-4, dipeptidyl peptidase-4; PD: Parkinson’s disease; OR, odds ratio; CI, confidence interval.

In the GTEx v8 dataset, eQTL information was available for 49 tissues and significant cis-eQTLs for *DPP4* were identified in four tissues (adrenal gland, lung, thyroid, and transverse colon). We therefore conducted MR analyses specifically for these four tissues. 49 tissues were as follows: adipose subcutaneous; adipose visceral (omentum); adrenal gland; artery aorta; artery coronary; artery tibial; brain amygdala; brain anterior cingulate cortex (BA24); brain caudate (basal ganglia); brain cerebellar hemisphere; brain cerebellum; brain cortex; brain frontal cortex (BA9); brain hippocampus; brain hypothalamus; brain nucleus accumbens (basal ganglia); brain putamen (basal ganglia); brain spinal cord (cervical C-1); brain substantia nigra; breast mammary tissue; cells – cultured fibroblasts; cells – ebv-transformed lymphocytes; colon sigmoid; colon transverse; esophagus gastroesophageal junction; esophagus mucosa; esophagus muscularis; heart atrial appendage; heart left ventricle; kidney cortex; liver; lung; minor salivary gland; muscle skeletal; nerve tibial; ovary; pancreas; pituitary; prostate; skin not sun-exposed (suprapubic); skin sun-exposed (lower leg); small intestine terminal ileum; spleen; stomach; testis; thyroid; uterus; vagina; whole blood.

**Supplementary Table 7. MR Analysis of Genetically Proxied DPP-4 Inhibitors Using Gene Expression Data in CD4+ T cells on the Risk of PD**

| **Cell Types** | **No. of SNPs** | **Men (n=12,054 + 11,999)^*^** | | **Women (n=7,384 + 12,389)^*^** | | **All (N=37,688 + 981,372)^*^** | |
| --- | --- | --- | --- | --- | --- | --- | --- |
|  |  | **OR (95% CI)** | ***P*-value** | **OR (95% CI)** | ***P*-value** | **OR (95% CI)** | ***P*-value** |
| memory regulatory T cells | 1 | 1.099 (1.022-1.175) | 0.016 | 0.98 (0.898-1.063) | 0.635 | 1.058 (0.976-1.140) | 0.18 |
| T helper 2 (TH2) cells | 1 | 1.097 (1.022-1.172) | 0.016 | 0.98 (0.899-1.062) | 0.635 | 1.057 (0.976-1.138) | 0.18 |

^*^ Numbers of cases and controls, respectively.

Abbreviations: MR, Mendelian randomization; DPP-4, dipeptidyl peptidase-4; PD: Parkinson’s disease; OR, odds ratio; CI, confidence interval.

**Supplementary Table 8. Mediation MR of Genetically Proxied DPP-4 Inhibition on the PD Risk via Type 2 Diabetes**

| **Exposure** | **Outcome** | **Men** | | | **Women** | | | **All** | | |
| --- | --- | --- | --- | --- | --- | --- | --- | --- | --- | --- |
|  |  | **No. of SNPs** | **OR (95% CI)** | ***P*-value** | **No. of SNPs** | **OR (95% CI)** | ***P*-value** | **No. of SNPs** | **OR (95% CI)** | ***P*-value** |
| **DPP-4** | **T2DM** |  |  |  |  |  |  |  |  |  |
| Gene Expression (IV_eQTL_) |  | - | - | - | - | - | - | 8 | **1.14 (1.01-1.28)** | **0.030** |
| Protein Level (IV_pQTL_) |  | - | - | - | - | - | - | 28 | **1.08 (1.03-1.14)** | **0.004** |
| **T2DM Risk** | **PD** | 23 | 1.03 (0.96-1.12) | 0.394 | 12 | 1.01 (0.92-1.11) | 0.856 | 155 | 1.03 (0.96-1.1) | 0.460 |
| **DPP-4^*^** | **PD** |  |  |  |  |  |  |  |  |  |
| Gene Expression (IV_eQTL_) |  |  |  |  |  |  |  |  |  |  |
| - T2DM associated (*p* < 0.05) |  | 2 | 2.2 (0.99-4.85) | 0.052 | 2 | 1.04 (0.53-2.01) | 0.914 | 2 | 1.46 (0.67-3.19) | 0.340 |
| - T2DM unassociated (*p* > 0.05) |  | 5 | 2.25 (1.34-3.8) | **0.002** | 6 | 1.49 (0.88-2.49) | 0.134 | 6 | 1.97 (1.12-3.48) | **0.019** |
| Protein Level (IV_pQTL_) |  |  |  |  |  |  |  |  |  |  |
| - T2DM associated (*p* < 0.05) |  | 4 | 1.39 (1.1-1.76) | **0.005** | 4 | 1.02 (0.79-1.32) | 0.875 | 5 | 1.15 (0.78-1.68) | 0.485 |
| - T2DM unassociated (*p* > 0.05) |  | 20 | 1.39 (1.11-1.75) | **0.005** | 20 | 1.17 (0.91-1.5) | 0.231 | 23 | 1.37 (1.08-1.74) | **0.009** |

* IVs were selected according to the results of single-SNP MR analysis on the T2DM risk.

Abbreviations: MR, Mendelian randomization; DPP-4, dipeptidyl peptidase-4; PD: Parkinson’s disease; OR, odds ratio; CI, confidence interval.

**Supplementary Table 9.** **Colocalization Analyses between Expression/Protein Quantitative Trait Loci and PD Risk**

| **IV** | **Sex** | **PP_H_0_** | **PP_H_1_** | **PP_H_2_** | **PP_H_3_** | **PP_H_4_** |
| --- | --- | --- | --- | --- | --- | --- |
| IV_eQTL_ | Men | 0.000 | 0.564 | 0.000 | 0.033 | 0.403 |
|  | Women | 0.000 | 0.954 | 0.000 | 0.034 | 0.012 |
|  | All | 0.000 | 0.908 | 0.000 | 0.075 | 0.017 |
| IV_pQTL_ | Men | 0.000 | 0.563 | 0.000 | 0.034 | 0.402 |
|  | Women | 0.000 | 0.952 | 0.000 | 0.036 | 0.012 |
|  | All | 0.000 | 0.863 | 0.000 | 0.121 | 0.016 |

^a^ Numbers of cases and controls, respectively.

Abbreviations: PD: Parkinson’s disease; IV, instrumental variable; eQTL, expression quantitative trait loci; pQTL, protein QTL; PP, posterior probability.

**STROBE-MR checklist of recommended items to address in reports of Mendelian randomization studies**^1^ ^2^

| **Item No.** | **Section** | **Checklist item** | **Page No.** | **Relevant text from manuscript** |
| --- | --- | --- | --- | --- |
| 1 | **TITLE and ABSTRACT** | Indicate Mendelian randomization (MR) as the study’s design in the title and/or the abstract if that is a main purpose of the study | 1 | Repurposing DPP4 Inhibitor for Parkinson’s Disease Prevention: A Drug-Target Mendelian Randomization Study |
|  | **INTRODUCTION** |  |  |  |
| 2 | **Background** | Explain the scientific background and rationale for the reported study. What is the exposure? Is a potential causal relationship between exposure and outcome plausible? Justify why MR is a helpful method to address the study question | 3 | Recent studies have identified promising therapeutic targets, with epidemiological evidence suggesting potential protective effects of anti-diabetic drugs, specifically glucagon-like peptide-1 receptor agonists (GLP-1RA) and dipeptidyl peptidase-4 inhibitors (DPP-4Is), on PD risk. Notably, GLP-1RAs demonstrated beneficial effects in phase 2 clinical trials for PD. Although a recent phase 3 trial of GLP1-RA on PD failed to meet its primary endpoint, it demonstrated favorable trends across secondary outcomes. Moreover, both GLP-1RAs and DPP-4Is have been associated with neuroprotective effects in other neurodegenerative diseases, including Alzheimer’s disease. Although the mechanism of DPP-4Is partly overlap with GLP-1RA, such as increasing incretin action, but its potential for PD management remains unconfirmed, as no clinical trials have been completed to date. |
| 3 | **Objectives** | State specific objectives clearly, including pre-specified causal hypotheses (if any). State that MR is a method that, under specific assumptions, intends to estimate causal effects | 4 | This study aims to evaluate the potential for repurposing DPP-4Is for preventing PD using a drug-target MR approach. |
|  | **METHODS** |  |  |  |
| 4 | **Study design and data sources** | Present key elements of the study design early in the article. Consider including a table listing sources of data for all phases of the study. For each data source contributing to the analysis, describe the following: |  |  |
|  | a) | Setting: Describe the study design and the underlying population, if possible. Describe the setting, locations, and relevant dates, including periods of recruitment, exposure, follow-up, and data collection, when available. | 4 | - In constructing the genetic IV for DPP-4, we employed the eQTL results from the eQTLGen consortium (phase 1), which incorporated 37 eQTL data from 31,684 samples of predominantly European ancestry - we also constructed genetic instruments related to plasma protein levels using data from a recent genome-wide association study (GWAS) of proteome in the UK Biobank. |
|  | b) | Participants: Give the eligibility criteria, and the sources and methods of selection of participants. Report the sample size, and whether any power or sample size calculations were carried out prior to the main analysis | 5 | We utilized data from the International Parkinson's Disease Genomics Consortium (IPDGC) which conducted GWAS on 37,688 PD patients and 981,372 controls, predominantly of European ancestry. |
|  | c) | Describe measurement, quality control and selection of genetic variants | 4, 5 | - We only extracted significant cis-eQTL loci located within ± 200 kb of the gene boundary, applying Bonferroni correction (p-value < 0.05/number of markers). From these data, we included SNPs with no linkage disequilibrium (r2 < 0.1) to generate the IVs. - We identified significant cis-pQTLs (within ± 200 kb) with p-values < 0.05/number of markers. |
|  | d) | For each exposure, outcome, and other relevant variables, describe methods of assessment and diagnostic criteria for diseases | NA | Not applicable, since this study solely utilized previously published research findings. |
|  | e) | Provide details of ethics committee approval and participant informed consent, if relevant | 8 | This study was exempt from Institutional Review Board (IRB) approval from Seoul National University (IRB No. E2105/003-010) since it solely used publicly available summary-level data from eQTL, pQTL, and GWAS data, none of which contain identifiable personal information. All studies used in this research obtained participant consent and ethical approval from their respective ethics review boards. |
| 5 | **Assumptions** | Explicitly state the three core IV assumptions for the main analysis (relevance, independence and exclusion restriction) as well assumptions for any additional or sensitivity analysis | 6 | - We evaluated three core IV assumptions (relevance, independence, and exclusion restriction assumptions) as follows. To ascertain the robustness of each IV (relevance assumption), we calculated their F statistics using the following formula:  - The independence assumption that the SNPs used as instruments for the exposure (DPP4 inhibition) are not associated with confounders was not evaluated, since we used the summary-level data. However, IVs specifically mimicking a drug target gene usually consist of very small number of SNPs (within ± 200 kb of DPP4 in this study), thereby violation of second assumption is unlikely. The exclusion restriction assumption (no pleiotropy) was evaluated with the MR-Egger analysis. |
| 6 | **Statistical methods: main analysis** | Describe statistical methods and statistics used |  |  |
|  | a) | Describe how quantitative variables were handled in the analyses (i.e., scale, units, model) | 5 | Both eQTL and pQTL results were derived using normalized data and |
|  | b) | Describe how genetic variants were handled in the analyses and, if applicable, how their weights were selected | 5 | beta estimates of each SNP on this normalized value were taken in our MR analysis. |
|  | c) | Describe the MR estimator (e.g. two-stage least squares, Wald ratio) and related statistics. Detail the included covariates and, in case of two-sample MR, whether the same covariate set was used for adjustment in the two samples | 5 | MR analysis was conducted using the inverse-variance weighted (IVW) method or a Wald ratio if there was only one SNP in the IV, |
|  | d) | Explain how missing data were addressed | NA | Not applicable, since this study solely utilized previously published research findings. |
|  | e) | If applicable, indicate how multiple testing was addressed | NA | Not applicable, since this study did not include multiple testing analysis. |
| 7 | **Assessment of assumptions** | Describe any methods or prior knowledge used to assess the assumptions or justify their validity |  |  |
| 8 | **Sensitivity analyses and additional analyses** | Describe any sensitivity analyses or additional analyses performed (e.g. comparison of effect estimates from different approaches, independent replication, bias analytic techniques, validation of instruments, simulations) | 6 | We assessed for heterogeneity and pleiotropy in all MR analyses. Upon detecting heterogeneity using Cochran’s Q statistic (p < 0.05), outliers were removed for re-analysis utilizing the ‘RadialMR’ software (p < 0.05). We employed the MR-Egger estimates when horizontal pleiotropy was detected (p <0.05). We also performed the MR-Steiger directionality test22 to confirm the direction of causality between DPP4 expression or protein levels and PD. Statistical power was calculated with mRnd (https://shiny.cnsgenomics.com/mRnd/) for each MR analysis.24 Analysis of IV robustness was further tested by employing varied LD clumping thresholds (r2 < 0.3, 0.2, 0.05, and 0.01), in addition to the initial 0.1. |
| 9 | **Software and pre-registration** |  |  |  |
|  | a) | Name statistical software and package(s), including version and settings used | 5 | utilizing the ‘TwoSampleMR’ package (v0.5.8) in R. |
|  | b) | State whether the study protocol and details were pre-registered (as well as when and where) | NA | Not applicable, since this study solely utilized previously published research findings. |
|  | **RESULTS** |  |  |  |
| 10 | **Descriptive data** |  |  |  |
|  | a) | Report the numbers of individuals at each stage of included studies and reasons for exclusion. Consider use of a flow diagram | NA | We performed two-sample MR analysis with summary-level GWAS data. We specified the numbers included in each GWAS study in the 'Methods' section, page 5. |
|  | b) | Report summary statistics for phenotypic exposure(s), outcome(s), and other relevant variables (e.g. means, SDs, proportions) | NA |  |
|  | c) | If the data sources include meta-analyses of previous studies, provide the assessments of heterogeneity across these studies | NA | We utilized the previously published meta-analysis where the heterogeneity was appropriately handled. |
|  | d) | For two-sample MR:  i.  Provide justification of the similarity of the genetic variant-exposure associations between the exposure and outcome samples | 4, 5  NA | **Exposure:** In constructing the genetic IV for DPP4, we employed the eQTL results from the eQTLGen consortium (phase 1), which incorporated 37 eQTL data from 31,684 samples of predominantly European ancestry.  **Outcome:** We utilized data from the International Parkinson's Disease Genomics Consortium (IPDGC) which conducted GWAS on 37,688 PD patients and 981,372 controls, predominantly of European ancestry.  Because the exposure datasets (eQTLGen and UK Biobank-based pQTL) and outcome datasets (PD GWAS excluding UK Biobank participants) were derived from largely independent sources, the risk of sample overlap is minimal. |
|  |  | ii.  Provide information on the number of individuals who overlap between the exposure and outcome studies |  |  |
| 11 | **Main results** |  |  |  |
|  | a) | Report the associations between genetic variant and exposure, and between genetic variant and outcome, preferably on an interpretable scale | 9 | From the MR analyses of these instruments on the risk of PD, both IVs consistently increased the risk of PD, suggesting the drugs inhibiting DPP4 may have a protective effect against PD. |
|  | b) | Report MR estimates of the relationship between exposure and outcome, and the measures of uncertainty from the MR analysis, on an interpretable scale, such as odds ratio or relative risk per SD difference | 9 | The effect sizes diminished in accordance with an increase in the biological distance between genetic variants and their targets: odds ratios (ORs) and 95% confidence intervals (CIs) using IVeQTL and IVpQTL were 1.78 [1.21-2.61], p = 0.003 and 1.21 [1.02-1.45], p = 0.032, respectively (Table 1). |
|  | c) | If relevant, consider translating estimates of relative risk into absolute risk for a meaningful time period | NA | Since the genetic study for the exposure variable used the normalized gene expression or protein value, we could not interpret the unit. |
|  | d) | Consider plots to visualize results (e.g. forest plot, scatterplot of associations between genetic variants and outcome versus between genetic variants and exposure) |  | Figure 2, Supplementary Figure 2 and 5 |
| 12 | **Assessment of assumptions** |  |  |  |
|  | a) | Report the assessment of the validity of the assumptions | 9 | As shown in the directed acyclic graph in Supplementary Fig. 1, the first (relevance) and third assumptions (exclusion restriction) are satisfied as F statistics (>10) and the results of pleiotropy test show (Supplementary Table 1). |
|  | b) | Report any additional statistics (e.g., assessments of heterogeneity across genetic variants, such as *I^2^*, Q statistic or E-value) |  | Supplementary Table 1 |
| 13 | **Sensitivity analyses and additional analyses** |  |  |  |
|  | a) | Report any sensitivity analyses to assess the robustness of the main results to violations of the assumptions | 9 | As shown in the directed acyclic graph in Supplementary Fig. 1, the first (relevance) and third assumptions (exclusion restriction) are satisfied as F statistics (>10) and the results of pleiotropy test show (Supplementary Table 1). |
|  | b) | Report results from other sensitivity analyses or additional analyses | 9 | Plots for sensitivity analyses including results from diverse MR methods, single SNP and leave-one-out analysis (stratified for sex and overall) were presented in Supplementary Fig. 2-4 and 5-7, for IVeQTL, and IVpQTL, respectively. These analyses consistently supported significant associations between DPP4 inhibition and overall PD, with the associations primarily driven by men. |
|  | c) | Report any assessment of direction of causal relationship (e.g., bidirectional MR) | 10 | Results of the MR-Steiger directionality test indicated the direction of causal effects of DPP4 on PD is true (p < 0.001; Supplementary Table 2). |
|  | d) | When relevant, report and compare with estimates from non-MR analyses | NA | Our effect estimates were derived from eQTL/pQTL data, where normalized values were used. Therefore, the comparison of estimates from other analyses are not applicable. |
|  | e) | Consider additional plots to visualize results (e.g., leave-one-out analyses) |  | Supplementary Figure 3, 4, 6, and 7 |
|  | **DISCUSSION** |  |  |  |
| 14 | **Key results** | Summarize key results with reference to study objectives | 12 | This study suggests that DPP-4Is could be effective in preventing PD in men, supporting their potential prioritization for male diabetic patients at higher risk of PD. Our genetic proxies of DPP-4, derived from both gene expression and protein data, consistently showed significant associations. Additionally, our findings revealed sex differences suggesting distinct relationships between DPP-4 and PD. |
| 15 | **Limitations** | Discuss limitations of the study, taking into account the validity of the IV assumptions, other sources of potential bias, and imprecision. Discuss both direction and magnitude of any potential bias and any efforts to address them | 15, 16 | Our study has several limitations. First, while we propose a potential effect of DPP-4Is on PD and its related traits, the scope of this study does not include the mechanism of this potential repurposing. Additionally, a drug-target MR study cannot fully replicate clinical trials, as it mimics low-dose lifelong exposures rather than reflecting high-dose short-term drug use in trials. Notably, the effect sizes from our analysis are estimated per unit increase of normalized gene expression or protein levels and do not reflect the actual dose of DPP-4Is. Genetic instruments using specific target proteins of drugs may not account for complex network between other proteins. Third, we used eQTL and pQTL data generated from sex-combined analyses, which may not fully capture sex-specific genetic effects. However, in our separate MR analyses using sex-stratified pQTL results with the UK Biobank dataset, the results (men: OR 2.47 [1.47-4.13], p = 6.2e-4; women: OR 1.04 [0.54-1.98], p = 0.912) were largely consistent with those derived from the sex-combined data, suggesting that the observed associations are unlikely to be driven solely by sex-related differences in genetic regulation. Fourth, our use of DPP-4 expression and protein levels as instruments may not fully capture the functional effects of DPP-4 inhibition. To address this, we conducted additional analyses using type 2 diabetes as a functional proxy, which suggested that the association with PD is unlikely to be mediated through glycemic pathways. Fifth, the absence of significant associations in women may reflect insufficient statistical power and should therefore be interpreted with caution. This could be due to the smaller sample size in female datasets, as well as the relatively weaker effect sizes observed in women. Finally, our results are limited to the European population and not fully validated in non-European populations. Caution should be taken when generalizing these findings to other populations. |
| 16 | **Interpretation** |  |  |  |
|  | a) | Meaning: Give a cautious overall interpretation of results in the context of their limitations and in comparison with other studies | 16 | In conclusion, this study highlights the protective role of DPP-4Is in male PD using genetic instruments for this drug target. Notably, the sex differences observed indicate a varying impact of DPP-4Is between males and females. Our findings support the potential of DPP-4Is as a promising therapeutic option in the prevention of PD in men. |
|  | b) | Mechanism: Discuss underlying biological mechanisms that could drive a potential causal relationship between the investigated exposure and the outcome, and whether the gene-environment equivalence assumption is reasonable. Use causal language carefully, clarifying that IV estimates may provide causal effects only under certain assumptions | 13, 14 | - Another possible explanation for the observed sex-specific effects of DPP-4 inhibition on PD risk could be the underlying phenotypic differences between males and females in PD manifestation.  - We also performed a mediation analysis to assess whether the effect of DPP-4 on PD risk is mediated through T2DM. While DPP-4 showed a suggestive association with T2DM as expected, we found no evidence that T2DM itself increases PD risk, consistent with previous MR report (36), and the main association between DPP-4 and PD was primarily driven by SNPs not associated with T2DM. These findings suggest that the observed DPP-4 and PD link is independent of T2DM. We further explored whether this association might be explained by altered GLP-1 signaling. However, our GLP1R-based drug-target MR showed no significant association with PD risk. This may reflect that receptor expression or protein abundance does not necessarily correspond to receptor activation status (37), as also observed in our analysis showing a null association with T2DM (data not shown). |
|  | c) | Clinical relevance: Discuss whether the results have clinical or public policy relevance, and to what extent they inform effect sizes of possible interventions | 12 | This study suggests that DPP-4Is could be effective in preventing PD in men, supporting their potential prioritization for male diabetic patients at higher risk of PD. |
| 17 | **Generalizability** | Discuss the generalizability of the study results (a) to other populations, (b) across other exposure periods/timings, and (c) across other levels of exposure | 16 | Finally, our results are limited to the European population and not fully validated in non-European populations. Caution should be taken when generalizing these findings to other populations. |
|  | **OTHER INFORMATION** |  |  |  |
| 18 | **Funding** | Describe sources of funding and the role of funders in the present study and, if applicable, sources of funding for the databases and original study or studies on which the present study is based | 17 | This research was supported by the National Research Foundation of Korea (NRF) grant funded by the Korea government (Ministry of Science and ICT) (2021R1C1C2011327 to J.-Y.L and RS-2023-00217595 to D.G.P.), Basic Science Research Program through the NRF funded by the Ministry of Education (RS-2023-00245246 to D.G.P.), and the Ministry of Food and Drug Safety (23212MFDS202 to J.S.). |
| 19 | **Data and data sharing** | Provide the data used to perform all analyses or report where and how the data can be accessed, and reference these sources in the article. Provide the statistical code needed to reproduce the results in the article, or report whether the code is publicly accessible and if so, where | 17 | We thank all contributing consortia and research groups for providing analysis data and making them publicly available. This study utilized the publicly accessible eQTL data from the eQTLGen consortium (data available at https://www.eqtlgen.org/phase1.html) and the GTEx consortium v8 (https://gtexportal.org/home/downloads/adult-gtex/qtl), and pQTL data from the UK Biobank Pharma Proteomics Project (UKB-PPP) with data available at https://www.synapse.org/Synapse:syn51365303. For the sex-stratified pQTL analysis, we accessed the UK Biobank Resource under Application Number 87860. For the GWAS result of PD, we used the data from the GWAS catalog (https://www.ebi.ac.uk/gwas/studies/GCST009324). Sex-stratified PD data were obtained from the IPDGC (https://pdgenetics.org/resources). For the replication dataset, we used the meta-analysis of PD results from the UK Biobank and FinnGen studies. For the T2DM GWAS, we used the data from DIAGRAM (for sex-stratified GWAS) and DIAMANTE consortium (for overall T2DM), accessible at https://www.diagram-consortium.org/downloads.html. We acknowledge all consortia and research groups who generated and made these data publicly available. |
| 20 | **Conflicts of Interest** | All authors should declare all potential conflicts of interest | 18 | All authors declare that they have no additional financial relationships or competing interests to disclose. |

This checklist is copyrighted by the Equator Network under the Creative Commons Attribution 3.0 Unported (CC BY 3.0) license.

1. Skrivankova VW, Richmond RC, Woolf BAR, Yarmolinsky J, Davies NM, Swanson SA, et al. Strengthening the Reporting of Observational Studies in Epidemiology using Mendelian Randomization (STROBE-MR) Statement. JAMA. 2021;under review.

2. Skrivankova VW, Richmond RC, Woolf BAR, Davies NM, Swanson SA, VanderWeele TJ, et al. Strengthening the Reporting of Observational Studies in Epidemiology using Mendelian Randomisation (STROBE-MR): Explanation and Elaboration. BMJ. 2021;375:n2233.
